# Quercetin Alleviates Lipopolysaccharide-Induced Inflammatory Response in Bovine Mammary Epithelial Cells by Suppressing TLR4/NF-κB Signaling Pathway

**DOI:** 10.3389/fvets.2022.915726

**Published:** 2022-07-04

**Authors:** Maocheng Jiang, Ziyao Lv, Yinghao Huang, Zhiqiang Cheng, Zitong Meng, Tianyu Yang, Qi Yan, Miao Lin, Kang Zhan, Guoqi Zhao

**Affiliations:** ^1^College of Animal Science and Technology, Institute of Animal Culture Collection and Application, Yangzhou University, Yangzhou, China; ^2^Chia Tai Tianqing Pharmaceutical Group Co., Ltd., Nanjing, China; ^3^Institutes of Agricultural Science and Technology Development, Yangzhou University, Yangzhou, China; ^4^Joint International Research Laboratory of Agriculture and Agri-Product Safety, The Ministry of Education of China, Yangzhou University, Yangzhou, China

**Keywords:** quercetin, bovine mammary epithelial cell, oxidative stress, barrier function, inflammatory

## Abstract

Bovine mastitis is one of the most common clinical diseases in dairy cows, causing huge economic losses to the dairy industry. Quercetin is an important flavonoid existing in many food resources, which has attracted widespread attention as a potential anti-inflammatory and antioxidant. However, the molecular mechanism of quercetin on inflammatory responses and oxidative stress in bovine mammary epithelial cells (BMECs) induced by lipopolysaccharide (LPS) remains unknown. The objective of this study was to investigate the effects of quercetin on inflammation responses, oxidative stress, and barrier function of BMEC induced by LPS. Our results showed that BMEC viability was not affected by treatment with 50 and 100 μg/ml of quercetin and 1 μg/ml of LPS compared with control group. The results of oxidative stress indicators and related genes of barrier function indicated that 100 μg/ml of quercetin effectively protected the BMECs from damage of oxidative and barrier induced by 1 μg/ml of LPS. Moreover, the messenger RNA (mRNA) expressions of pro-inflammatory cytokines TNF-α, IL-1β, IL-6, and chemokines CXCL2, CXCL5, CCL5, and CXCL8 were markedly decreased in the LPS-treated bovine retinal endothelial cells (BRECs) with 100 μg/ml of quercetin relatively to LPS alone. More importantly, the mRNA expressions of toll-like receptor 4 (TLR4), CD14, myeloid differential protein-2 (MD2), and myeloid differentiation primary response protein (MyD88) genes involved in TLR4 signal pathway were significantly attenuated by the addition of quercetin in LPS-treated BMEC, suggesting that quercetin can inhibit the TLR4 signal pathway. In addition, immunocytofluorescence showed that quercetin significantly inhibited the nuclear translocation of NF-κB p65 in BMEC induced by LPS. Therefore, the protective effects of quercetin on inflammatory responses in LPS-induced BMEC may be due to its ability to suppress the TLR4-mediated NF-κB signaling pathway. These findings suggest that quercetin can be used as an anti-inflammatory reagent to treat mastitis induced by exogenous or endogenous LPS release.

## Introduction

Bovine mastitis is a transmissible disease that decreases milk quality and yield (1). Furthermore, mastitis increases the culling rate, resulting in considerable economic losses to dairy farming worldwide (2). Mastitis has a direct negative impact on cow's health status and associated consequences on the productivity and welfare of the cow (3, 4). Regardless of *Staphylococcus aureus* and *Escherichia coli* both being considered major mastitis pathogens, the mammary environment is known to be a reservoir of *Staphylococcus*, while *E. coli* is mainly considered an environmental mastitis-causing bacteria (5). Lipopolysaccharide (LPS) is a major component of the outer membrane in gram-negative bacteria which induces a strong immune response in the mammary gland (6, 7). Innate immune responses initiated by LPS in bovine mammary epithelial cells (BMECs) have been widely used in the study of mastitis *in vitro* (8). In general, pattern recognition receptors such as the Toll-like receptors (TLRs) can recognize LPS and activate intracellular signaling pathways, leading to the pro-inflammatory responses (9). Previous research has shown that TLRs play a critical role in the induction of innate immune and inflammatory responses (10). The LPS-LBP-CD14-MD2 complex elicits a pro-inflammatory response by TLR4-mediated activation of NF-κB signal pathway to induce the expression of pro-inflammatory cytokines and chemokines (11). These inflammatory mediators are then involved in cellular homeostasis and have systemic effects (12). In the past decade, antibiotics is represented one of the major means of mastitis treatment (5). However, this treatment often leads to the presence of antibiotic residue (13) and bacterial drug resistance (14). Thus, the development of novel alternative drugs is essential for the prevention and treatment of mastitis.

Quercetin is a flavonoid, which is a secondary metabolite of plant synthesis, and found in many fruits and vegetables (15). Quercetin has antioxidant and anti-inflammatory properties and modulates cell apoptosis, properties potentially of value in the treatment or prevention of mastitis (16). A previous research has shown that quercetin has an anti-cancer ability by inhibiting of growth of cells (17). In some studies, quercetin has been shown to reduce oxidative damage induced by inflammatory cytokines, leading to improving the survival and function of cells (18). It has been reported that quercetin suppresses the expression of IL-1β, IL-6, and TNF-α in LPS-treated human gingival fibroblasts (15). In addition, quercetin not only possesses strong antioxidant properties through free radical scavenging but also reduces inflammation and inhibits cell proliferation and angiogenesis (19). However, it is not clear whether quercetin can protect BMECs from inflammatory damage.

We hypothesized that quercetin can influence the inflammatory response of LPS-induced BMEC through the negative regulation of TLR signaling pathway. Therefore, this study explored the improvement effects of quercetin on LPS-induced mammary epithelial cell inflammation in dairy cows, and explored its possible mechanism, so as to provide a theoretical basis for further research and development in treating mastitis using quercetin-based novel alternative drugs.

## Materials and Methods

### Mammary Epithelial Cell Culture

The BMECs were sourced from the Institute of Animal Culture Collection and Application, Yangzhou University, China. The establishment was based on previous studies (20). The BMCEs were cultured in Dulbecco's modified Eagle medium (DMEM)/F12 medium (Gibco, Grand Island, NY, USA) supplemented with 10% fetal bovine serum (Gibco), 100 U/ml of penicillin, and 100 μg/ml of streptomycin (Sigma, 85886, USA). The culture was incubated at 37°C under 5% CO_2_.

### Effects of Quercetin and LPS on BMEC Viability

The BMEC viability was measured using the cell counting kit-8 (CCK-8; Vazyme, Nanjing, China). Quercetin (>95%) and LPS were purchased from Sigma-Aldrich (St. Louis, MO, USA). Quercetin was dissolved in dimethyl sulfoxide (DMSO) and DMEM/F12 medium to a stock solution of 200 μg/ml and subsequently passed through a.22-μm sterile filter. The BMECs were seeded into 96-well plates (1 × 10^4^ cells per well) for 12 h, and then treated with 1 μg/ml of LPS and different concentrations of quercetin (50, 100, 150, or 200 μg/ml) for 6 h. Subsequently, 10 μl of CCK8 was added into all of the experimental groups and incubated at 37°C, 5% CO_2_ for 3 h. The optical density (OD) values were acquired at 450 nm using a microplate reader.

### Antioxidant Analysis

The BMECs were added to 6-well culture plates and adjusted to 1 × 10^6^ cells/ml. The cells proliferated to about 80–90% and treated with LPS and quercetin. After 6 h of culture, these cells were collected. Total antioxidant capacity (TAC), malondialdehyde (MDA), glutonium peroxidase (GSH-Px), superoxide dismutase (SOD), and catalase (CAT) were measured in cell samples using the antioxidant related assay kit (Jiancheng Bioengineering Institute, Nanjing, China) according to the manufacturer's protocol. The TAC, MDA, GSH-Px, SOD, and CAT levels are expressed as U/mg.protein (prot) in relation to the cellular protein concentration.

### RNA Extraction, Reverse Transcription, and Real-Time PCR

Ribonucleic acid was extracted from BMEC using a TRIzol reagent (Takara, Code No. RR036A, China) according to the manufacturer's instructions, and the RNA concentrations were determined using an OD1000 instrument (One drop 1000, Nanjing, China). Then, RNA was reverse transcribed to complementary DNA (cDNA) using a standard reverse transcription kit (Takara, Tokyo, Japan) according to the instructions. The PCR reaction was performed with the SYBR^®^ Premix Ex Taq™ II Kit (Takara, Dalian, China). The relative gene expression was normalized by GAPDH using the 2^−Δ*ΔCt*^ method (21). All primers used are listed in Table 1 (synthesized in GENEWIZ Bioscience Co., Ltd., Suzhou, China).

**Table 1:** Primers for real-time quantitative PCR.

**Gene**	**Primer sequence, 5^′^-3^′^**	**Source**	**Size (bp)**
GAPDH	F: GGGTCATCATCTCTGCACCT R: GGTCATAAGTCCCTCCACGA	NM_001034034.2	176
IL-1β	F: CAGTGCCTACGCACATGTCT R: AGAGGAGGTGGAGAGCCTTC	NM_174093.1	209
IL-6	F: CACCCCAGGCAGACTACTTC R: TCCTTGCTGCTTTCACACTC	NM_173923.2	129
TNF-α	F: GCCCTCTGGTTCAGACACTC R: AGATGAGGTAAAGCCCGTCA	NM_173966.3	192
CXCL5	F: TGAGACTGCTATCCAGCCG R: AGATCACTGACCGTTTTGGG	NM_174300.2	193
CCL5	F: CTGCCTTCGCTGTCCTCCTGATG R: TTCTCTGGGTTGGCGCACACCTG	NM_175827	217
CCL2	F: GCTCGCTCAGCCAGATGCAA R: GGACACTTGCTGCTGGTGACTC	NM_174006	171
CXCL2	F: CCCGTGGTCAACGAACTGCGCTGC R: CTAGTTTAGCATCTTATCGATGATT	NM_174299.3	204
CXCL8	F: TGGGCCACACTGTGAAAAT R: TCATGGATCTTGCTTCTCAGC	NM_173925.2	136
ZO-1	F: TCTGCAGCAATAAAGCAGCATTTC R: TTAGGGCACAGCATCGTATCACA	XM_010817146.1	187
Occludin	F: GAACGAGAAGCGACTGTATC R: CACTGCTGCTGTAATGAGG	NM_001082433.2	122
Claudin 1	F: CGTGCCTTGATGGTGAT R: CTGTGCCTCGTCGTCTT	NM_001001854.2	102
Claudin 4	F: CTTCATCGGCAGCAACATC R: ACAACAGCACGCCAAACA	NM_001014391.2	191
TLR2	F: CAGGCTTCTTCTCTGTCTTGT R: CTGTTGCCGACATAGGTGATA	NM_174197.2	140
TLR4	F:GACCCTTGCGTACAGGTTGT R:GGTCCAGCATCTTGGTTGAT	NM_174198.6	103
CD14	F: CAGTATGCTGACACAATCAA R: AGTTCCTTGAGACGAGAGTA	NM_174008.1	122
MD2	F: GGAGAATCGTTGGGTCTGC R: GCTCAGAACGTATTGAAACAGGA	NM_001046517.1	92
MyD88	F: TCATTGAGAAGAGGTGCCGT R: TGGCTTGTACTTGATGGGGAT	NM_001014382.2	146
IRAK1	F: CCTCAGCGACTGGACATCCT R: GGACGTTGGAACTCTTGACATCT	NM_001040555.1	103
IRF3	F: TTGTGAACTCAGGAGTCAGG R: TGGGCTCAAGTCCATGTCAC	NM_001029845.3	125

*F, forward; R, reverse*.

### Immunocytofluorescence Analysis

The cells were cultured on a 8-well chamber slide (Thermo Scientific, Lab-TekTMII, Code No. 154534, NY, USA) at a density of.5 × 10^4^ cells/well. They were grown to ~80% confluency and treated with 100 μg/ml of quercetin and 5 μg/ml of LPS. The coverslips were then washed three times with PBS and fixed with acetone:methanol = 1:1 for 30 min at room temperature, washed three times with PBS for 3 min each time, and then subjected to antigen retrieval with EDTA-Na_2_ (95°C, 5 min), after which they were rewashed. Following treatment with.1% Triton X-100 (Sigma-Aldrich, St. Louis, MO) and washing with PBS three times, the cells were blocked with a 3% horse serum-containing blocking buffer. After further washes, ERK1/2 (p44/42) rabbit mAb (1:800; Cell Signaling Technology, Shanghai, China), phosphorylation-ERK1/2 (p-p44/42) rabbit mAb (1:1,000; Cell Signaling Technology, Shanghai, China), NF-κB (p65) rabbit mAb (1:500; Cell Signaling Technology, Shanghai, China), and phosphorylation-NF-κB (p-p65) rabbit mAb (1:500; Cell Signaling Technology, Shanghai, China) were then added and incubated overnight at 4°C, and then exposed to goat anti-rabbit IgG conjugated with Cy3 (Beyotime Biotechnology Inc.) for 30 min. The cultured cells were washed with PBS three times and then 4'6-diamidino-2-phenylindole (DAPI) staining solution was added for 7 min. Finally, an immunofluorescence microscopy was performed using a fluorescence microscopy (Olympus, Tokyo, Japan).

### Statistical Analysis

The data are presented as the means and the SEM. The significant differences were determined by the one-way ANOVA and the Tukey's multiple comparison tests. The analysis was conducted using the SPSS Statistics software, version 20.0 (IBM Corp., Armonk, NY, USA). The statistical significance was defined at *P* < 0.05 with highly significant values at *P* < 0.01; trends were declared at 0.05 < *P* < 0.1.

## Results

### Effects of Quercetin on Viability of BMECs

**Figure 1:**
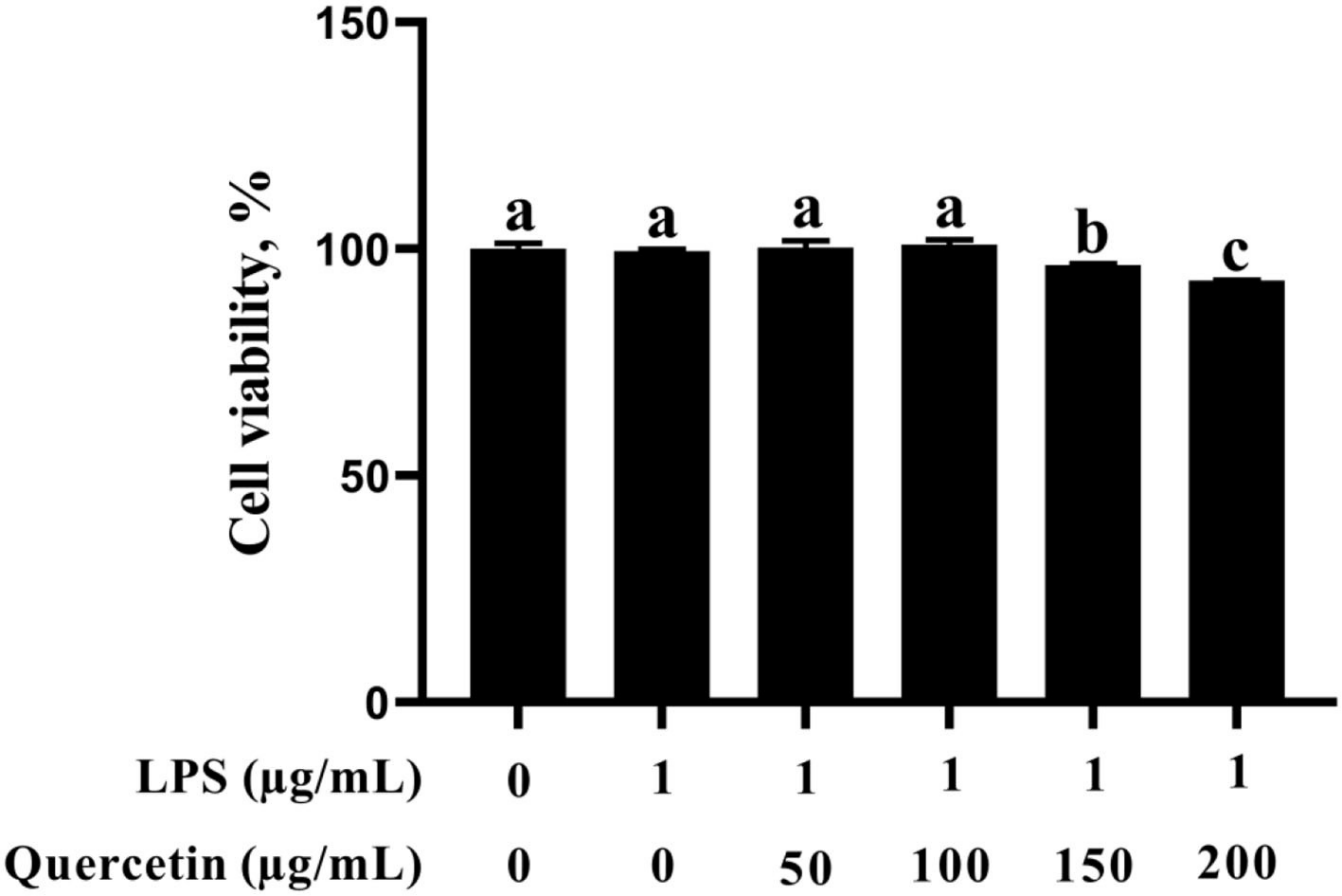
Effects of quercetin on cell viability in lipopolysaccharide (LPS)-induced bovine mammary epithelial cells (BMECs). BMECs were treated with 1 μg/ml of LPS and different concentrations of quercetin (50, 100, 150, or 200 μg/ml) for 6 h. The data from the control group were used to normalize the data of each treatment group. Data presented are mean ± SEM (*n* = 4). Different lowercase letters in the bar chart indicate significant differences (*P* < 0.05).

In this study, the BMEC viability was not altered after stimulation with 1 μg/ml of LPS (Figure 1). In addition, BMECs with 1 μg/ml of LPS treatment were subjected to 50, 100, 150, and 200 μg/ml of quercetin for 6 h. As was observed from Figure 1, BMEC viability was not affected by treatment with 50 and 100 μg/ml of quercetin and 1 μg/ml of LPS compared with the control group. However, 150 and 200 μg/ml of quercetin remarkably reduced the cell viability of LPS-treated BMECs (*P* < 0.05). Thus, 1 μg/ml of LPS and 100 μg/ml of quercetin were selected as the subsequent treatment concentrations.

### Effects of Quercetin on Oxidative Properties of BMECs Stimulated by LPS

**Figure 2:**
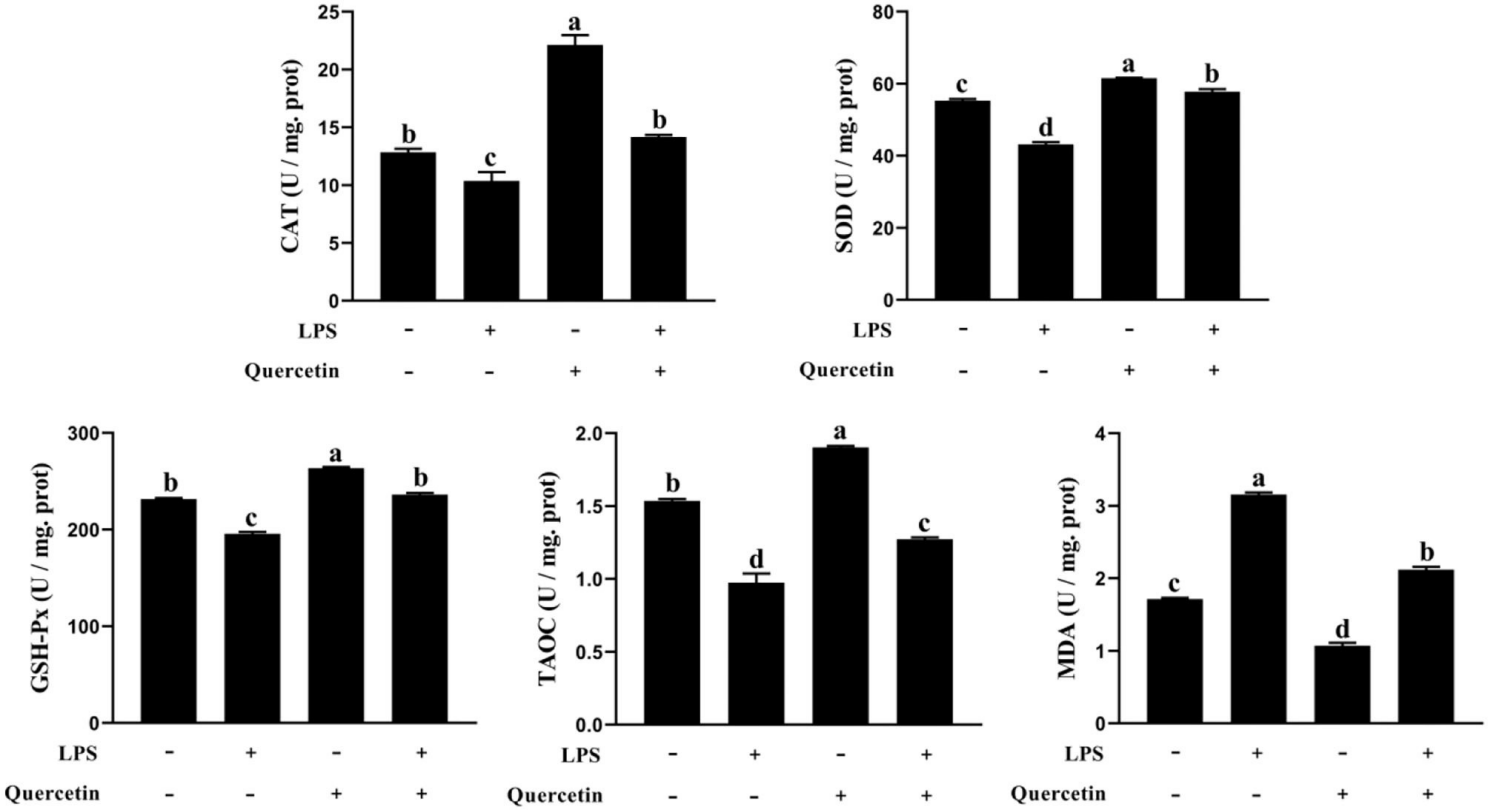
Effects of 100 μg/ml of quercetin on oxidative properties of 1 μg/ml of LPS-treated BMECs. BMECs were treated with 1 μg/ml of LPS and 100 μg/ml of quercetin. After 6 h of culture, the cells were collected. Catalase (CAT), superoxide dismutase (SOD), glutonium peroxidase (GSH-Px), total anti-oxidant capacity (TAC), and malondialdehyde (MDA) were measured in cell samples using the antioxidant related assay Kit. Data presented are mean ± SEM (*n* = 3). Different lowercase letters in the bar chart indicate significant differences (*P* < 0.05).

We also investigated the ability of quercetin to protect the BMECs induced by LPS from the oxidative stress. The TAOC, SOD, CAT, and GSH-Px contents were decreased in LPS-treated groups than the control group (*P* < 0.05, Figure 2). However, TAOC, SOD, CAT, and GSH-Px levels were increased in BMECs treated by quercetin alone compared with the control group (*P* < 0.05). More importantly, quercetin enhanced (*P* < 0.05) the level of TAOC, SOD, CAT, and GSH-Px in BMEC stimulated by LPS relatively to LPS alone. Furthermore, the MDA content in the BMECs after LPS treatment was significantly increased compared to that in the control, whereas treatment with quercetin significantly reduced the MDA content in LPS-treated BMCE (*P* < 0.05). These findings indicated that quercetin contributed to the ability of antioxidant in LPS-induced BMEC.

### Effects of Quercetin on Barrier Function of BMECs Induced by LPS

**Figure 3:**
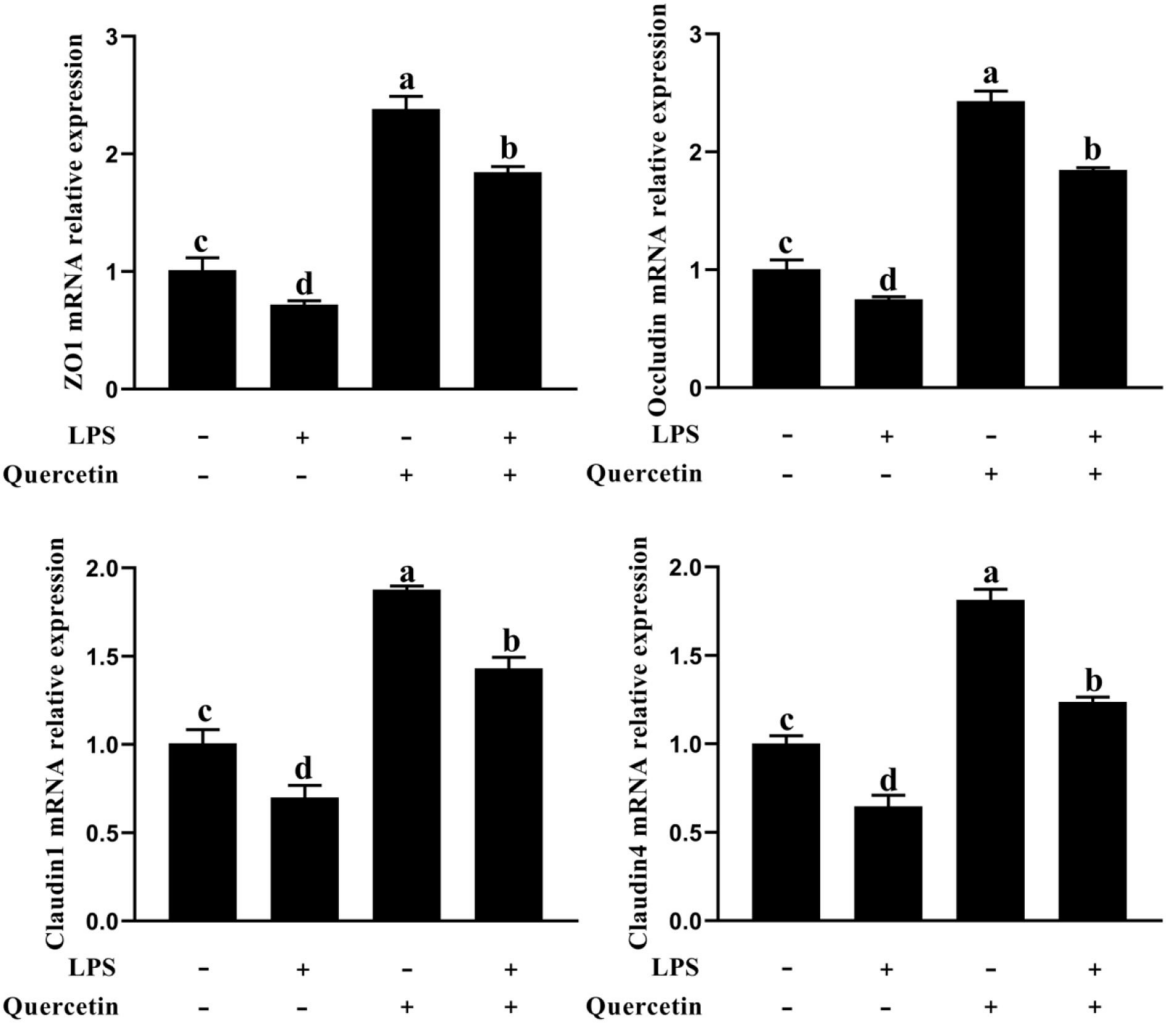
Effects of quercetin on barrier function in LPS-induced BMECs. BMECs were treated with 100 μg/ml of quercetin in the presence of 1 μg/ml of LPS for 6 h; messenger RNA (mRNA) expressions of zo-1, occluding, claudin 1, and claudin 4 in BMECs were assayed by qRT-PCR. Data presented are mean ± SEM (*n* = 3). Different lowercase letters in the bar chart indicate significant differences (*P* < 0.05).

To determine whether quercetin has the protective effects on barrier function for LPS-treated BMEC, we analyzed the messenger RNA (mRNA) expression of zo-1, occluding, claudin 1, and claudin 4 by a qRT-PCR analysis. As shown in Figure 3, the mRNA expressions of zo-1, occluding, claudin 1, and claudin 4 were significantly downregulated in all groups treated with LPS compared to the control group (*P* < 0.05). In contrast, the mRNA expressions of zo-1, occluding, claudin 1, and claudin 4 were considerably upregulated in BMEC induced by LPS and with 100 μg/ml of quercetin compared with the LPS-treated group (*P* < 0.05, Figure 3). These results demonstrated that quercetin has protective effects in LPS-induced BMEC barrier disruption.

### Effects of Quercetin on Pro-Inflammation Factors of BMECs Induced by LPS

**Figure 4:**
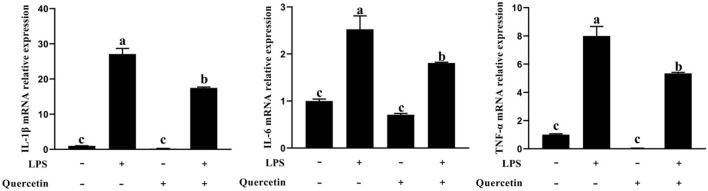
Effects of quercetin on the mRNA expression of pro-inflammatory cytokines (IL-1β, IL-6, and TNF-α) in LPS-induced BMECs. BMECs were treated with 100 μg/ml of quercetin in the presence of 1 μg/ml of LPS for 6 h. The mRNA expressions of IL-1β, IL-6, and TNF-α in BMEC were assayed by qRT-PCR. Data presented are mean ± SEM (*n* = 3). Different lowercase letters in the bar chart indicate significant differences (*P* < 0.05).

To examine the effects of quercetin on the production of pro-inflammatory cytokines in LPS-stimulated BMECs, genes involved in the pro-inflammatory responses were analyzed by qRT-PCR (Figure 4). In comparison with the control group, the mRNA expressions of IL-1β, IL-6, and TNF-α were upregulated in BMECs stimulated with LPS. However, the mRNA expressions of TNF-α, IL-1β, and IL-6 were significantly decreased in LPS-treated BMECs with quercetin compared with the LPS-treated group (*P* < 0.05, Figure 4). These findings indicated that quercetin can inhibit the expression of pro-inflammatory cytokines in LPS-induced BMECs.

### Effects of Quercetin on Immune Response of BMECs Induced by LPS

**Figure 5:**
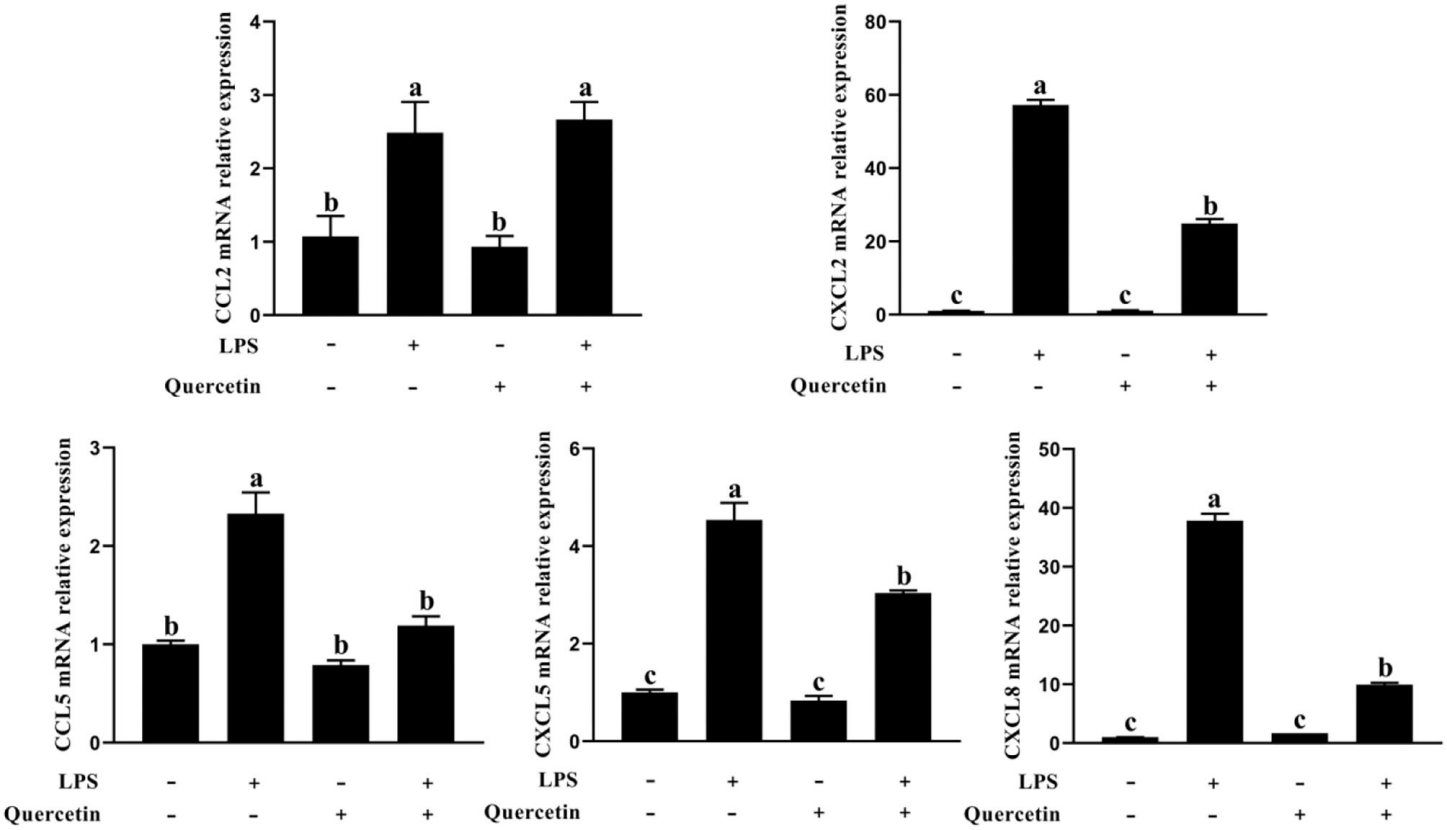
Effects of quercetin on the mRNA expression of chemokines in LPS-induced BMECs. BMECs were treated with 100 μg/ml of quercetin in the presence of 1 μg/ml of LPS for 6 h. The mRNA expressions of CCL2, CCL5, CXCL2, CXCL5, and CXCL8 in BMEC were assayed by qRT-PCR. Data presented are mean ± SEM (*n* = 3). Different lowercase letters in the bar chart indicate significant differences (*P* < 0.05).

The BMECs with LPS treatment significantly enhanced the mRNA expressions of CCL2, CCL5, CXCL2, CXCL5, and CXCL8 genes compared with the unstimulated cells (*P* < 0.05, Figure 5). On the contrary, the expressions of CCL5, CXCL2, CXCL5, and CXCL8 genes involved in immune responses were markedly downregulated in the treatment group with 100 μg/ml of quercetin in the presence of 1 μg/ml of LPS, relative to BMECs treated with 1 μg/ml of LPS (*P* < 0.05, Figure 5). These results revealed that quercetin relieved LPS-induced immune responses of BMECs.

### Effects of Quercetin on MRNA Expression of the TLR4 Signaling Pathway of BMECs Induced by LPS

**Figure 6:**
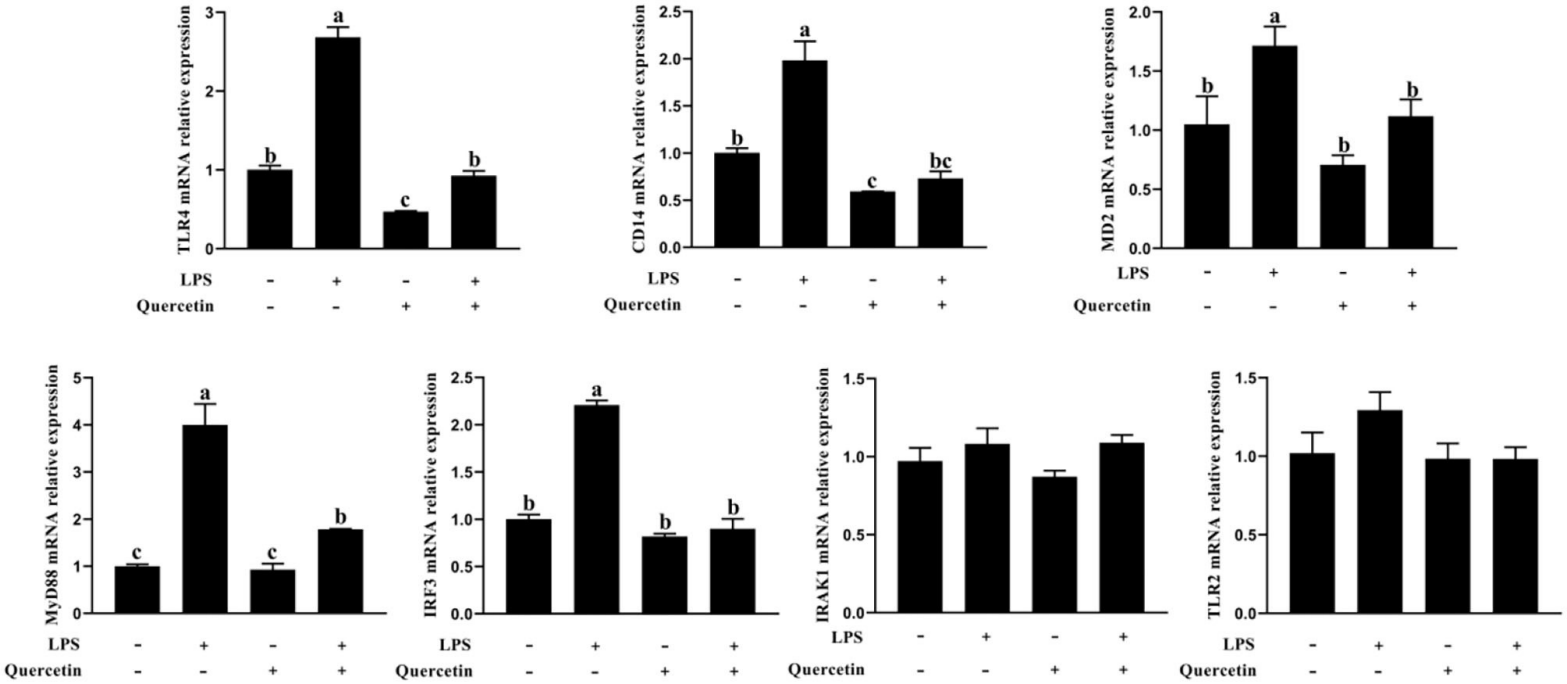
Effects of quercetin on the TLR4 signaling pathway in LPS-induced BMECs. BMECs were treated with 100 μg/ml of quercetin in the presence of 1 μg/ml of LPS for 6 h. The mRNA expressions of TLR4, TLR2, CD14, MD2, MyD88, IRF3, and IRAK1 in BMEC were assayed by qRT-PCR. Data presented are mean ± SEM (*n* = 3). Different lowercase letters in the bar chart indicate significant differences (*P* < 0.05).

The expressions of TLR4, CD14, MD2, MyD88, interleukin 1 receptor associated kinase 1 (IRAK1), and IRF3 genes involved in TLR4 signaling pathway were measured by a qRT-PCR analysis. As shown in Figure 6, the mRNA levels of TLR4, CD14, MD2, MyD88, and IRF3 were significantly increased after treatment with LPS, but were significantly reduced in LPS-induced BMECs with 100 μg/ml of quercetin treatment (*P* < 0.05, Figure 6). The quercetin and LPS have no profound effects in the expression of TLR2 and IRAK1 (*P* > 0.05, Figure 6). These data demonstrated that quercetin alleviates LPS-induced pro-inflammatory responses in BMECs by suppressing the TLR4 signaling pathway.

### Effects of Quercetin on NF-κB and ERK1/2 Signaling Pathway of BMECs Induced by LPS

**Figure 7:**
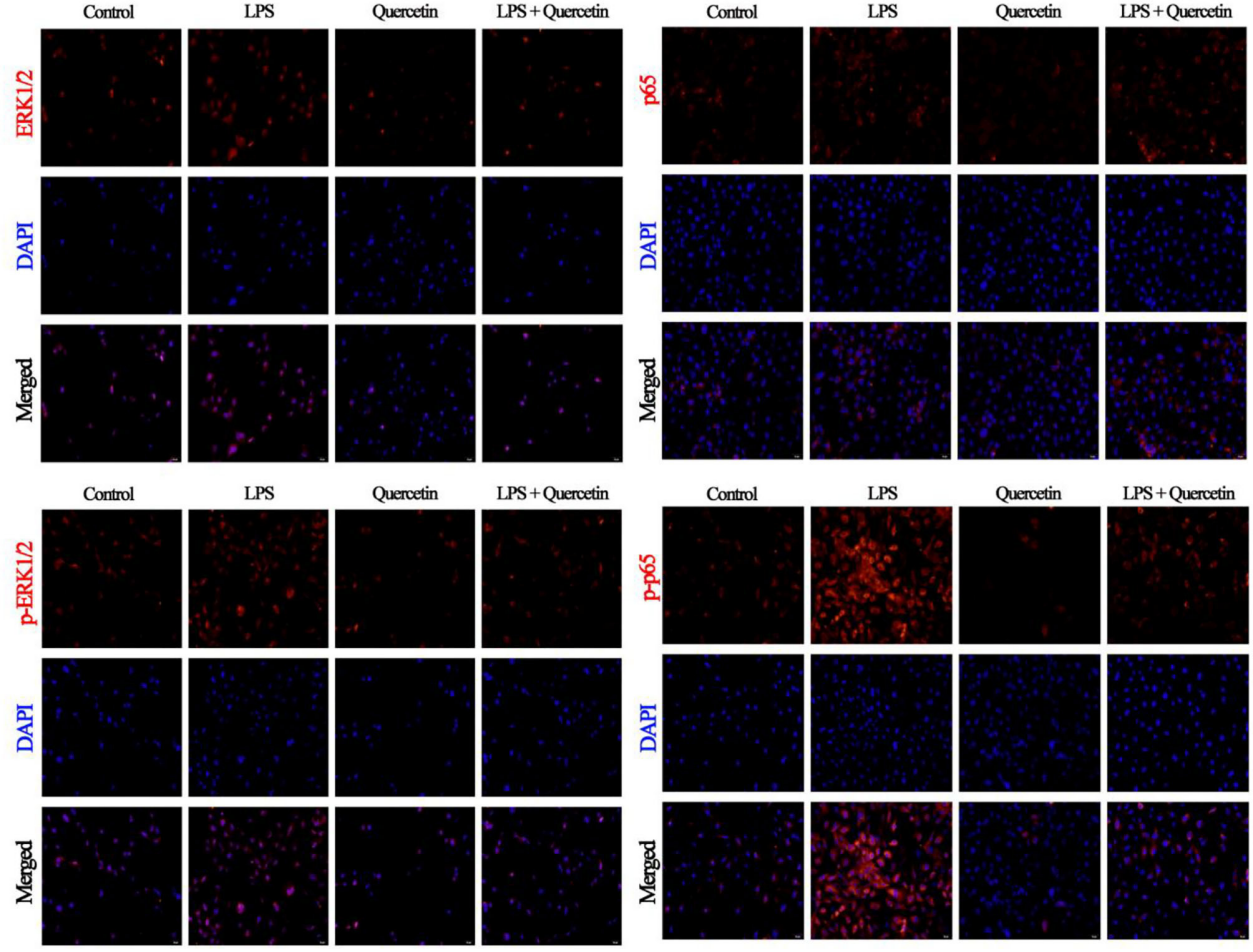
Effects of quercetin on NF-κB p65 and ERK1/2 signaling pathway in LPS-induced BMECs. BMECs were treated with 100 μg/ml of quercetin in the presence of 1 μg/ml of LPS for 6 h. The immunofluorescence for NF-κB p65 and ERK1/2 (red) was performed, and the nuclear dye 4′, 6-diamidino-2-phenylindole (DAPI; blue) was used. Scale bar = 50 μm.

The results of immunofluorescence indicated that LPS activates the ERK1/2 pathway through TLR4 in BMECs leading to increased levels of ERK1/2, but we did not detect any alteration in the phosphorylation level of ERK1/2 following quercetin treatment, compared with the level of ERK1/2 (Figure 7). Moreover, we also found that LPS treatment significantly increased the nuclear entry of p-p65 in BMEC, whereas quercetin treatment inhibitory effects on the nuclear entry of p-p65 (Figure 7).

## Discussion

The BMEC is thought to play an important role against invasion of pathogenic microorganisms (22). Previous studies suggest that the mammary gland suffered to large amounts of LPS stimulation with pathogenic microorganisms, and sustained LPS can exacerbate the mammary gland inflammatory responses (23). The LPS is a major component of the outer membrane in gram-negative bacteria, which induces a strong immune response in the mammary gland (7).

The presence of high concentrations of LPS can lead to oxidative stress caused by the imbalance of cell redox (24). Excessive oxidative stress activates immune cells to induce the inflammatory responses cascade. Available evidence suggests an interaction between inflammatory response and oxidative stress (25). On one hand, inflammatory cells produce reactive oxygen species (ROS) to participate in oxidative stress; on the other hand, ROS can lead to increasing expression of inflammatory cytokines (26). In addition, the BMEC was involved in the formation of the blood–milk barrier that plays an important role in protecting mammary gland function in mammals (5). A previous research showed that mastitis can alter blood-milk barrier permeability, and the composition of mastitic milk is considerably changed, such as the increased Somatic Cell Count (SCC) (27). This blood–milk barrier could be due to structural alterations in tight junction protein. Overuse of antibiotics causes antibiotics to lose effectiveness over time (28). Therefore, the development of alternative, non-toxic agents for the resolution LPS-induced pro-inflammatory responses and oxidative stress is an urgent need for improving the barrier function and mounting the innate immune responses to promote the resolution of LPS infection.

At present, plant extracts containing anti-inflammatory and anti-inflammatory compounds are an effective and safe strategy to clinical treat bovine mastitis. Quercetin has been reported to have an anti-inflammatory and antioxidant effects (29, 30). Previous reports have shown that quercetin protects gastric epithelial cells from oxidative damage both *in vitro* and *in vivo* (31) and reduces paraquat-induced oxidative damage by regulating the expression of antioxidant genes in cells (32). Xiong et al. (15) reported that quercetin ameliorated LPS-induced inflammation in human gingival fibroblasts (HGFs) by activating PPAR-γ and suppressing the activation of NF-κB. Many studies showed promising therapeutic potential for quercetin in treating inflammatory diseases (33–36). Importantly, recent studies indicate that flavonoids exert protective effects on the health of humans and animals (37). However, the regulation mechanisms of quercetin on anti-inflammatory, antioxidant, and barrier function are still not clear in LPS-induced BMECs at present. Thus, in this study, we investigated the protective effects of quercetin on the anti-inflammatory, antioxidant, and barrier functions in LPS-induced BMECs *in vitro*. Previous studies have shown that LPS reacts with TLR4, triggering the activation of the NF-κB signal pathway and the production of ROS to regulate the expression of the activities of antioxidant enzymes (38). In this study, we further examined the SOD, GSH-Px, TAOC, MDA, and CAT contents in LPS-induced BMECs. The SOD, GSH-Px, TAOC, and CAT were significantly enhanced in LPS-induced BMECs with quercetin treatment relative to LPS alone. However, MDA was significantly reduced by addition of quercetin. As expected, these indicators of antioxidant enzymes were reduced by LPS treatment, which is consistent with the present results (39). Therefore, our results demonstrated that quercetin enhanced the content of SOD, CAT, GSH-Px, and TAOC in LPS-induced BMECs and reduced the MDA content, eventually alleviating the pro-inflammatory responses of BMECs.

We next sought to explore the function of quercetin in LPS-induced injury of barrier function in BMECs. The inflammatory cytokines produced by cells in response to LPS reduce epithelial barrier integrity, leading to exacerbating the epithelial damage and inflammation (40). Prior work has demonstrated that quercetin enhanced the intestinal barrier function through upregulation of Cldn4 in Caco-2 cells (41). Previous studies have shown that important determinants of epithelial barrier function and paracellular permeability are the maintenance of intercellular tight junction expression (42). We further analyzed the epithelial tight-junction protein marker (zo-1, occluding, claudin 1, and claudin 4) mRNA expression by qRT-PCR. The mRNA expressions of zo-1, occluding, claudin 1, and claudin 4 were significantly downregulated in all groups treated with LPS compared to the control group and indicated impairment of tight junction integrity. However, quercetin can promote the mRNA expression of tight junction protein. This result suggested that quercetin has protective effects against barrier disruption of LPS-induced BMECs.

To explore the anti-inflammatory activity of quercetin, we determined its action in LPS-treated BMECs. TLR4 is a pattern recognition receptor (43). TLR4 can recognize LPS and then activate the NF-κB signaling pathway, which stimulates p-p65 protein accumulation in the nucleus and the subsequent induction of inflammatory responses, promoting the secretion of inflammatory cytokines, such as TNF-α, IL-6, and IL-1β (44). Others similarly found that TLR4 activates MyD88 and then induces the activation of NF-κB and MAPK signaling pathways to increase the release of pro-inflammatory cytokines (45). When pathogens invade, BMECs can trigger the activation of the immune response through the induction of chemokines and adhesion molecules (46). Cytokines and chemokines play important roles in orchestrating LPS-mediated immune responses (47). The main role of chemokines is to recruit a large number of immune cells to the point of tissue localization, and these cells secrete various active products, which are involved in immune damage and inflammatory response of the tissue. Our results showed that chemokines CXCL2, CXCL5, CCL5, and CXCL8 were significantly upregulated in the LPS group compared to the control group. This suggests that LPS can promote the expression of chemokines, and the increased expression of chemokines can further chemotactic immune cells to the mammary gland immune layer to process the LPS. In contrast, the chemokine expression was significantly decreased in the quercetin and LPS + quercetin groups relative to the LPS group. In addition, LPS stimulation significantly increased the mRNA expression of pro-inflammatory cytokines (TNF-α, IL-6, and IL-1β) in BMECs, whereas treatment with quercetin reversed this trend, indicating that quercetin did have the capacity to suppress LPS-induced inflammation responses in the BMECs. Similarly, the result of related genes of TLR4 signaling pathway indicated that the mRNA levels of TLR4, CD14, MD2, MyD88, and IRF3 were significantly increased after treatment with LPS, but were significantly reduced in LPS-induced BMECs with quercetin treatment. In addition, the results of immunofluorescence indicated that LPS stimulation significantly increased the nuclear entry of p-p65 in BMEC, whereas quercetin treatment inhibited effects on the nuclear entry of p-p65 in LPS-induced BMECs. These results demonstrated that quercetin can alleviate the inflammatory responses of BMECs induced by LPS *via* inhibiting TLR4-NF-κB signaling pathway. Meanwhile, these results suggest that quercetin may be beneficial for the treatment of mastitis.

## Conclusions

This study has proved that quercetin alleviates the oxidative stress and the damage of barrier function of BMECs induced by LPS *in vitro*. In addition, quercetin can reduce the inflammatory response in LPS-induced BMECs by inhibiting TLR4-NF-κB signal pathway. In conclusion, quercetin can inhibit the LPS-induced oxidative stress and protect the normal physiological function of BMECs *via* TLR4-NF-κB signaling pathways.

## Data Availability Statement

The original contributions presented in the study are included in the article/supplementary material, further inquiries can be directed to the corresponding author/s.

## Ethics Statement

The animal study was reviewed and approved by Animal Care and Use Committee of Yangzhou University, Yangzhou, China and Ethic code is DWLL-202011-201.

## Author Contributions

MJ and KZ designed the whole experiment, verified the validity of experiment, and checked the results. ZL, QY, and ML performed the experiment, including chemical analysis, and statistical analysis. GZ, TY, and YH worked on the manuscript. ZC, YH, and ZM participated in the experiment design and gave valuable advice. ZL revised the manuscript. All of the authors have read and approved the final version of this manuscript.

## Funding

This study was supported by the National Natural Science Foundation of China (No. 31972589), the earmarked fund for CARS36, and the Excellent Doctoral Dissertation Fund of Yangzhou University.

## Conflict of Interest

ZL was employed by Chia Tai Tianqing Pharmaceutical Group Co., Ltd. The remaining authors declare that the research was conducted in the absence of any commercial or financial relationships that could be construed as a potential conflict of interest.

## Publisher's Note

All claims expressed in this article are solely those of the authors and do not necessarily represent those of their affiliated organizations, or those of the publisher, the editors and the reviewers. Any product that may be evaluated in this article, or claim that may be made by its manufacturer, is not guaranteed or endorsed by the publisher.

## References

[1] Puerto MA Shepley E Cue RI Warner D Dubuc J Vasseur E. The hidden cost of disease: I. Impact of the first incidence of mastitis on production and economic indicators of primiparous dairy cows. J Dairy Sci. (2021) 104:7932–43. 10.3168/jds.2020-19584 33865582

[2] Aghamohammadi M Haine D Kelton DF Barkema HW Hogeveen H Keefe GP. Herd-level mastitis-associated costs on Canadian dairy farms. Front Vet Sci. (2018) 5:100. 10.3389/fvets.2018.00100 29868620 PMC5961536

[3] Melchior MB Vaarkamp H Fink-Gremmels J. Biofilms: a role in recurrent mastitis infections? Vet J. (2006) 171:398–407. 10.1016/j.tvjl.2005.01.006 16624706

[4] Viguier C Arora S Gilmartin N Welbeck K O'kennedy R. Mastitis detection: current trends and future perspectives. Trends Biotechnol. (2009) 27:486–93. 10.1016/j.tibtech.2009.05.004 19616330

[5] Hu XY Guo J Zhao CJ Jiang P Maimai T Li YY. The gut microbiota contributes to the development of *Staphylococcus aureus*-induced mastitis in mice. ISME J. (2020) 14:1897–910. 10.1038/s41396-020-0651-1 32341472 PMC7305118

[6] Schneider M Zimmermann AG Roberts RA Zhang L Swanson KV Wen HT. The innate immune sensor NLRC3 attenuates toll-like receptor signaling *via* modification of the signaling adaptor TRAF6 and transcription factor NF-kappa B. Nat Immunol. (2012) 13:823–31. 10.1038/ni.2378 22863753 PMC3721195

[7] Guo WJ Liu BR Yin YH Kan XC Gong Q Li YW. Licochalcone A protects the blood-milk barrier integrity and relieves the inflammatory response in LPS-induced mastitis. Front Immunol. (2019) 10:7. 10:287. 10.3389/fimmu.2019.00287 30858849 PMC6398509

[8] Wang YN Zhang X Wei ZK Wang JJ Zhang Y Shi MY. Platycodin D suppressed LPS-induced inflammatory response by activating LXR alpha in LPS-stimulated primary bovine mammary epithelial cells. Eur J Pharmacol. (2017) 814:138–43. 10.1016/j.ejphar.2017.07.037 28736281

[9] Vanaja SK Russo AJ Behl B Banerjee I Yankova M Deshmukh SD. Bacterial outer membrane vesicles mediate cytosolic localization of LPS and caspase-11 activation. Cell. (2016) 165:1106–19. 10.1016/j.cell.2016.04.015 27156449 PMC4874922

[10] Sabbah A Chang TH Harnack R Frohlich V Tominaga K Dube PH. Activation of innate immune antiviral responses by Nod2. Nat Immunol. (2010) 11:969. 10.1038/ni1010-969b 19701189 PMC2752345

[11] Citronberg JS Curtis KR White E Newcomb PA Newton K Atkinson C. Association of gut microbial communities with plasma lipopolysaccharide-binding protein (LBP) in pre-menopausal women. ISME J. (2018) 12:1631–41. 10.1038/s41396-018-0064-6 29434315 PMC6018759

[12] Wang X Antony V Wang Y Wu GJ Liang G. Pattern recognition receptor-mediated inflammation in diabetic vascular complications. Med Res Rev. (2020) 40:2466–84. 10.1002/med.21711 32648967

[13] Zhang S Shen YR Wu S Xiao YQ He Q Shi SR. The dietary combination of essential oils and organic acids reduces *Salmonella enteritidis* in challenged chicks. Poult Sci. (2019) 98:6349–55. 10.3382/ps/pez457 31393588 PMC8913765

[14] Shi SR Wu S Shen YR Zhang S Xiao YQ He X. Iron oxide nanozyme suppresses intracellular *Salmonella enteritidis* growth and alleviates infection *in vivo*. Theranostics. (2018) 8:6149–62. 10.7150/thno.29303 30613289 PMC6299686

[15] Xiong G Ji WS Wang F Zhang FX Xue P Cheng M. Quercetin inhibits inflammatory response induced by LPS from *Porphyromonas gingivalis* in human gingival fibroblasts *via* suppressing NF-kappa B signaling pathway. Biomed Res Int. (2019) 2019:1–10. 10.1155/2019/6282635 31531360 PMC6720363

[16] Wan H Wang YP Pan QY Chen X Chen SJ Li XH. Quercetin attenuates the proliferation, inflammation, and oxidative stress of high glucose-induced human mesangial cells by regulating the miR-485-5p/YAP1 pathway. Int J Immunopathol Pharmacol. (2022) 36:1–12. 10.1177/20587384211066440 35129398 PMC8832592

[17] Cheng SP Gao N Zhang Z Chen G Budhraja A Ke ZJ. Quercetin induces tumor-selective apoptosis through downregulation of Mcl-1 and activation of Bax. Clin Cancer Res. (2010) 16:5679–91. 10.1158/1078-0432.CCR-10-1565 21138867 PMC3069720

[18] Liu MY Zhang CJ Duan LX Luan QQ Li JL Yang AG. CsMYB60 is a key regulator of flavonols and proanthocyanidans that determine the colour of fruit spines in cucumber. J Exp Bot. (2019) 70:69–84. 10.1093/jxb/ery336 30256979 PMC6305189

[19] Ekstrom AM Serafini M Nyren O Wolk A Bosetti C Bellocco R. Dietary quercetin intake and risk of gastric cancer: results from a population-based study in Sweden. Ann Oncol. (2011) 22:438–43. 10.1093/annonc/mdq390 20688844 PMC3030468

[20] Zhan K Yang T Feng B Zhu X Chen Y Huo Y. The protective roles of tea tree oil extracts in bovine mammary epithelial cells and polymorphonuclear leukocytes. J Anim Sci Biotechnol. (2020) 11:62. 10.1186/s40104-020-00468-9 32549980 PMC7294674

[21] Li CM Li L Chen KL Wang YR Yang FX Wang GL. UFL1 alleviates lipopolysaccharide-induced cell damage and inflammation *via* regulation of the TLR4/NF-kappa B pathway in bovine mammary epithelial cells. Oxid Med Cell Longev. (2019) 2019:1–17. 10.1155/2019/6505373 30881595 PMC6387704

[22] Rainard P Riollet C. Mobilization of neutrophils and defense of the bovine mammary gland. Reprod Nutr Dev. (2003) 43:439–57. 10.1051/rnd:2003031 15005373

[23] Klaas IC Zadoks RN. An update on environmental mastitis: challenging perceptions. Transbound Emerg Dis. (2018) 65 Suppl 1:166–85. 10.1111/tbed.12704 29083115

[24] Clarke AJ Riffelmacher T Braas D Cornall RJ Simon AK. B1a B cells require autophagy for metabolic homeostasis and self-renewal. J Exp Med. (2018) 215:399–413. 10.1084/jem.20170771 29326381 PMC5789411

[25] Furman D Chang JL Lartigue L Bolen CR Haddad F Gaudilliere B. Expression of specific inflammasome gene modules stratifies older individuals into two extreme clinical and immunological states. Nat Med. (2017) 23:174–84. 10.1038/nm.4267 28092664 PMC5320935

[26] Huang W Wang GF Lu SE Kipen H Wang YD Hu M. Inflammatory and oxidative stress responses of healthy young adults to changes in air quality during the Beijing olympics. Am J Respir Crit Care Med. (2012) 186:1150–9. 10.1164/rccm.201205-0850OC 22936356 PMC3530204

[27] Cebron N Maman S Walachowski S Gausseres B Cunha P Rainard P. Th17-related mammary immunity, but not a high systemic Th1 immune response is associated with protection against *E. coli* mastitis. NPJ Vaccines. (2020) 5:108. 10.1038/s41541-020-00258-4 33298970 PMC7686320

[28] Zhang S Zhong G Shao D Wang Q Hu Y Wu TX. Dietary supplementation with *Bacillus subtilis* promotes growth performance of broilers by altering the dominant microbial community. Poult Sci. (2021) 100:100935. 10.1016/j.psj.2020.12.032 33652528 PMC7936199

[29] Li Y Yao JY Han CY Yang JX Chaudhry MT Wang SN. Quercetin, inflammation and immunity. Nutrients. (2016) 8:167. 10.3390/nu8030167 26999194 PMC4808895

[30] Liu W Zhang MJ Feng JQ Fan AQ Zhou YL Xu YJ. The influence of quercetin on maternal immunity, oxidative stress, and inflammation in mice with exposure of fine particulate matter during gestation. Int J Environ Res Public Health. (2017) 14:592. 10.3390/ijerph14060592 28574437 PMC5486278

[31] Hu XT Ding C Zhou N Xu C. Quercetin protects gastric epithelial cell from oxidative damage *in vitro* and *in vivo*. Eur J Pharmacol. (2015) 754:133–42. 10.1016/j.ejphar.2015.02.007 25701726

[32] Zerin T Kim YS Hong SY Song HY. Quercetin reduces oxidative damage induced by paraquat *via* modulating expression of antioxidant genes in A549 cells. J Appl Toxicol. (2013) 33:1460–7. 10.1002/jat.2812 22996356

[33] Warren CA Paulhill KJ Davidson LA Lupton JR Taddeo SS Hong MY. Quercetin may suppress rat aberrant crypt foci formation by suppressing inflammatory mediators that influence proliferation and apoptosis. J Nutr. (2009) 139:792. 10.3945/jn.109.104935 19056647 PMC2714375

[34] Kao TK Ou YC Raung SL Lai CY Liao SL Chen CJ. Inhibition of nitric oxide production by quercetin in endotoxin/cytokine-stimulated microglia. Life Sci. (2010) 86:315–21. 10.1016/j.lfs.2009.12.014 20060843

[35] Burmanczuk A Hola P Milczak A Piech T Kowalski C Wojciechowska B. Quercetin decrease somatic cells count in mastitis of dairy cows. Res Vet Sci. (2018) 117:255–9. 10.1016/j.rvsc.2018.01.006 29331686

[36] Batiha GE Beshbishy AM Ikram M Mulla ZS El-Hack MEA Taha AE. The pharmacological activity, biochemical properties, and pharmacokinetics of the major natural polyphenolic flavonoid: quercetin. Foods. (2020) 9:374. 10.3390/foods9030374 32210182 PMC7143931

[37] Xiao Y Zhang S Tong H Shi S. Comprehensive evaluation of the role of soy and isoflavone supplementation in humans and animals over the past two decades. Phytother Res. (2018) 32:384–94. 10.1002/ptr.5966 29193539

[38] Shen JB Yang MZ Ju DH Jiang H Zheng JP Xu ZH. Disruption of SM22 promotes inflammation after artery injury *via* nuclear factor kappa B activation. Circ Res. (2010) 106:1351–62. 10.1161/CIRCRESAHA.109.213900 20224039 PMC2896867

[39] Sun AZ Nie SJ Xing D. Nitric oxide-mediated maintenance of redox homeostasis contributes to NPR1-dependent plant innate immunity triggered by lipopolysaccharides. Plant Physiol. (2012) 160:1081–96. 10.1104/pp.112.201798 22926319 PMC3461531

[40] Wlodarska M Luo C Kolde R D'hennezel E Annand JW Heim CE. Indoleacrylic acid produced by commensal peptostreptococcus species suppresses inflammation. Cell Host Microbe. (2017) 22:25–37.e26. 10.1016/j.chom.2017.06.007 28704649 PMC5672633

[41] Chen H Hu YH Fang Y Djukic Z Yamamoto M Shaheen NJ. Nrf2 deficiency impairs the barrier function of mouse oesophageal epithelium. Gut. (2014) 63:711–9. 10.1136/gutjnl-2012-303731 23676441 PMC3883925

[42] Wen L Javed TA Yimlamai D Mukherjee A Xiao XW Husain SZ. Transient high pressure in pancreatic ducts promotes inflammation and alters tight junctions *via* calcineurin signaling in mice. Gastroenterology. (2018) 155:1250. 10.1053/j.gastro.2018.06.036 29928898 PMC6174093

[43] Holland WL Bikman BT Wang LP Yuguang G Sargent KM Bulchand S. Lipid-induced insulin resistance mediated by the proinflammatory receptor TLR4 requires saturated fatty acid-induced ceramide biosynthesis in mice. J Clin Investig. (2011) 121:1858–70. 10.1172/JCI43378 21490391 PMC3083776

[44] Lu YC Yeh WC Ohashi PS. LPS/TLR4 signal transduction pathway. Cytokine. (2008) 42:145–51. 10.1016/j.cyto.2008.01.006 18304834

[45] Wang JF Zhang X He XJ Yang B Wang H Shan XF. LPS-induced reduction of triglyceride synthesis and secretion in dairy cow mammary epithelial cells *via* decreased SREBP1 expression and activity. J Dairy Res. (2018) 85:439–44. 10.1017/S0022029918000547 30088470

[46] Qian C Cao XT. Regulation of Toll-like receptor signaling pathways in innate immune responses. Ann N Y Acad Sci. (2013) 1283:67–74. 10.1111/j.1749-6632.2012.06786.x 23163321

[47] Woodall MC Woodall BP Gao EH Yuan A Koch WJ. Cardiac fibroblast GRK2 deletion enhances contractility and remodeling following ischemia/reperfusion injury. Circ Res. (2016) 119:1116–27. 10.1161/CIRCRESAHA.116.309538 27601479 PMC5085864

